# Supplementary figures and images for: PAX6 protein in neuromasts of the lateral line system of salamanders (Eurycea)
Source: PLoS One. 2024 Aug 30;19(8):e0293163. doi: 10.1371/journal.pone.0293163 (PMC11364236; doi:10.1371/journal.pone.0293163)

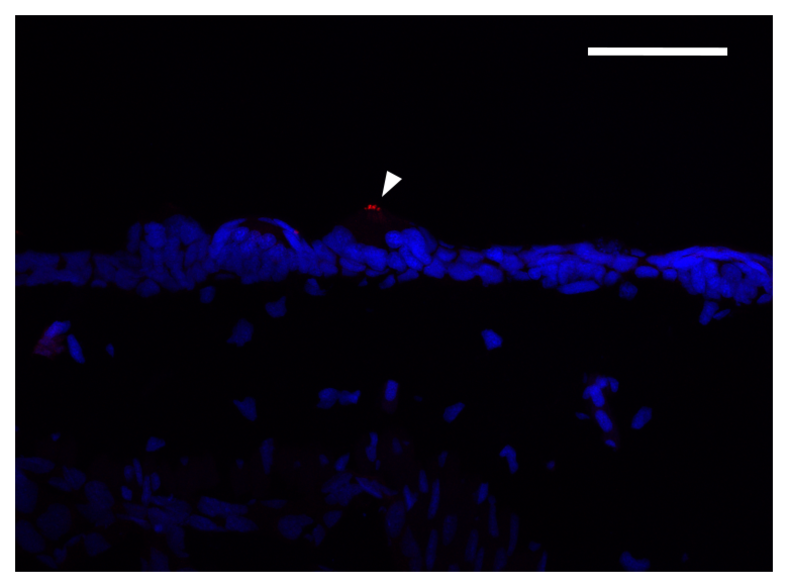

Supplement: S1 Fig — An arrowhead points to the PAX6 labeling (red) of apical projections of hair cells in the cross sectioned neuromast. The neuromast to the left, which was sectioned off-center (evident by the mantle cells extending over the apical surface), does not show PAX6 labeling. The scale bar in the image is 100 μm. Image represents Z-projections acquired using a 20x air objective with a numerical aperture of 0.75. (TIF) [file pone.0293163.s001.tif]

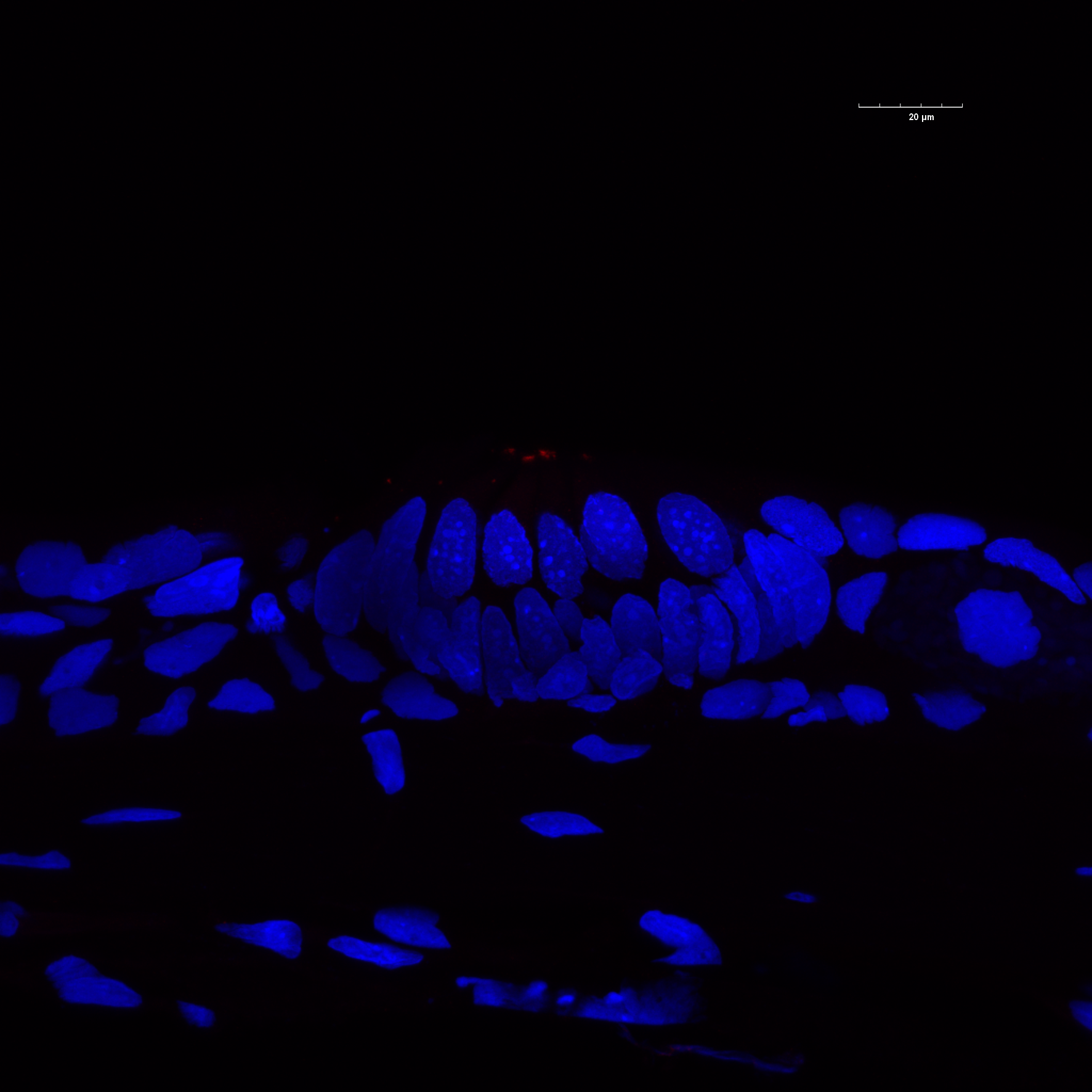

Supplement: S2 Fig — PAX6 (red) localizes to the apical appendages (arrowhead) of hair cells in the neuromast of an adult Texas blind salamander. The scale bar in the image is 20 μm, and the image represents a Z-projection acquired at the same magnification and settings as used in Fig 1(E,F). Nuclei are labeled blue with DAPI. (TIF) [file pone.0293163.s002.tif]

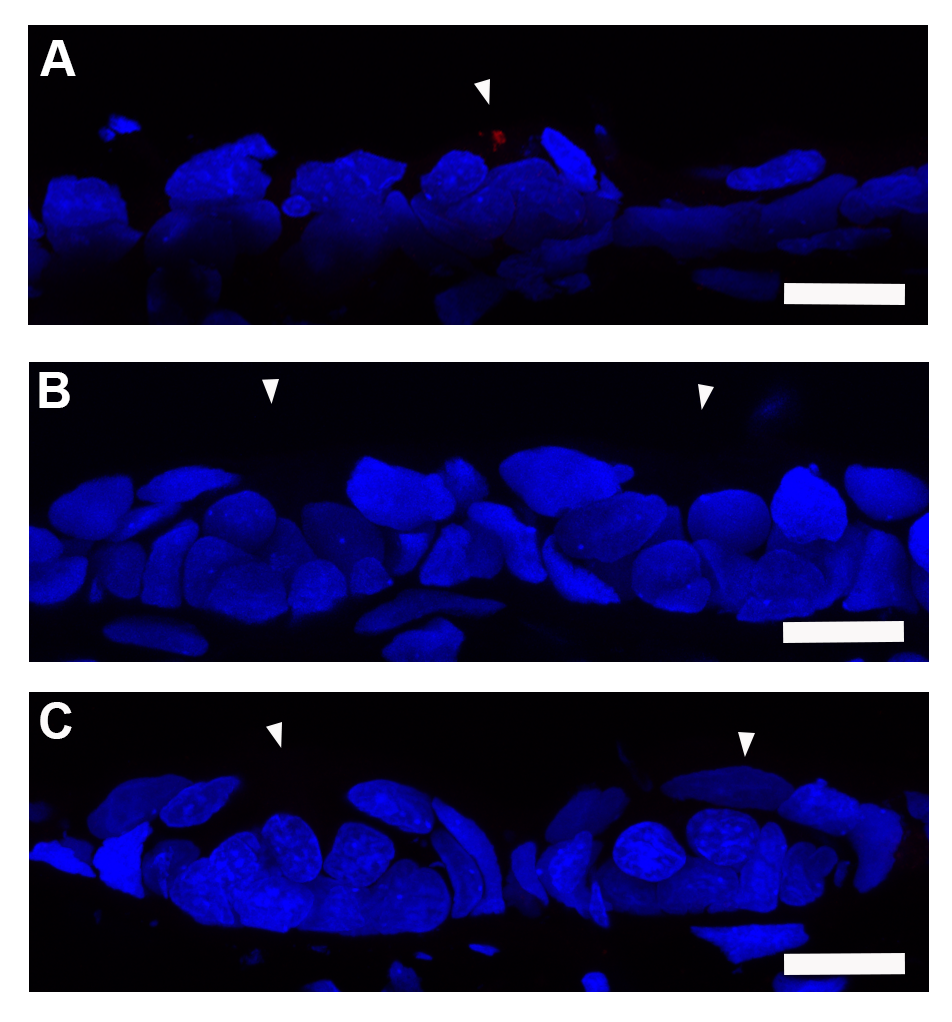

Supplement: S3 Fig — IHC images of E. rathbuni experimental (A) and negative control (B,C) slides. The PAX6 labeling in neuromasts of the experimental sections (A) is not detected in preadsorbed primary antibody sections (C) or the no primary antibody sections (D) serving as negative controls. Arrowheads point towards the apical appendages of hair cells in neuromasts. The scale bars in the images are 20 μm, and the images represent Z-projections acquired at the same magnification and settings. Nuclei are labeled blue with DAPI. (TIF) [file pone.0293163.s003.tif]

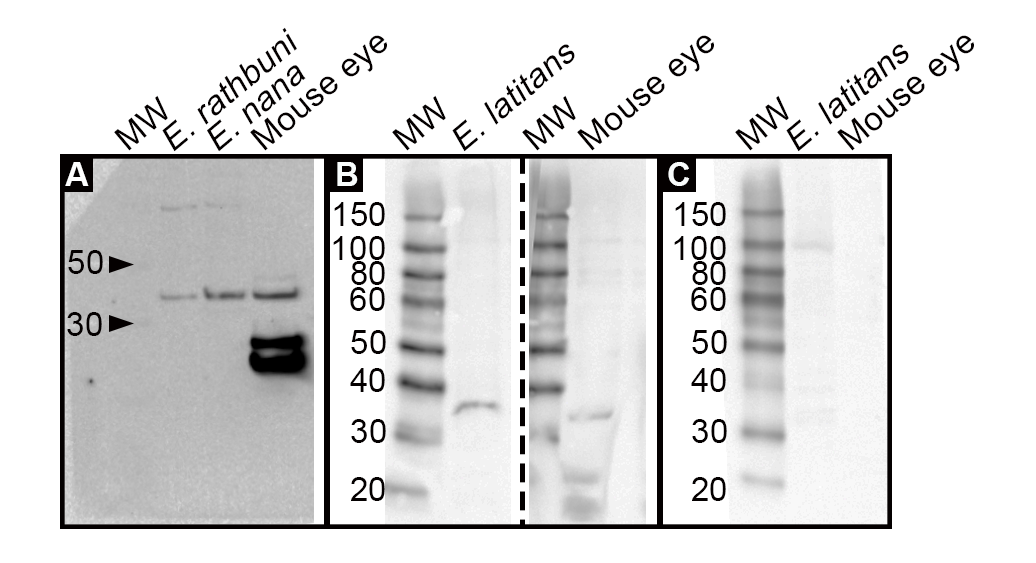

Supplement: S4 Fig — Tissue lysates were obtained from embryos of E. rathbuni, E. nana, and E. latitans as well as adult mouse eye to serve as a positive control. PagerRuler Plus (Thermo Fisher, cat #26619) [A] and SuperSignal (Thermo Fisher, cat # 84785) molecular weight markers [B,C] were used to estimate the molecular weight of labeled bands. A western analysis using our primary PAX6 antibody suggests that the antibody labels a protein of about 36 kDa in all four taxa. (A) Arrowheads are overlaid on each triangular notch cut into the membrane. E. rathbuni, E. nana, and mouse eye lysates have one labeled band in common at around 36 kDa. A broad, labeled region can be seen in the mouse eye lane between 20 kDa and 27 kDa. (B) E. latitans and mouse eye have one labelled band in common at around 36 kDa. The dotted line indicates where replicate lanes were cropped from the image. The lanes to the right of the dotted line were flipped horizontally to match the lane order of those to the left. (C) Preadsorbed antibody does not bind to the 36 kDa protein from E. latitans or mouse eye. Labeling of the lower molecular weight bands in mouse eye is also absent following preadsorption of the primary antibody. (TIF) [file pone.0293163.s004.tif]

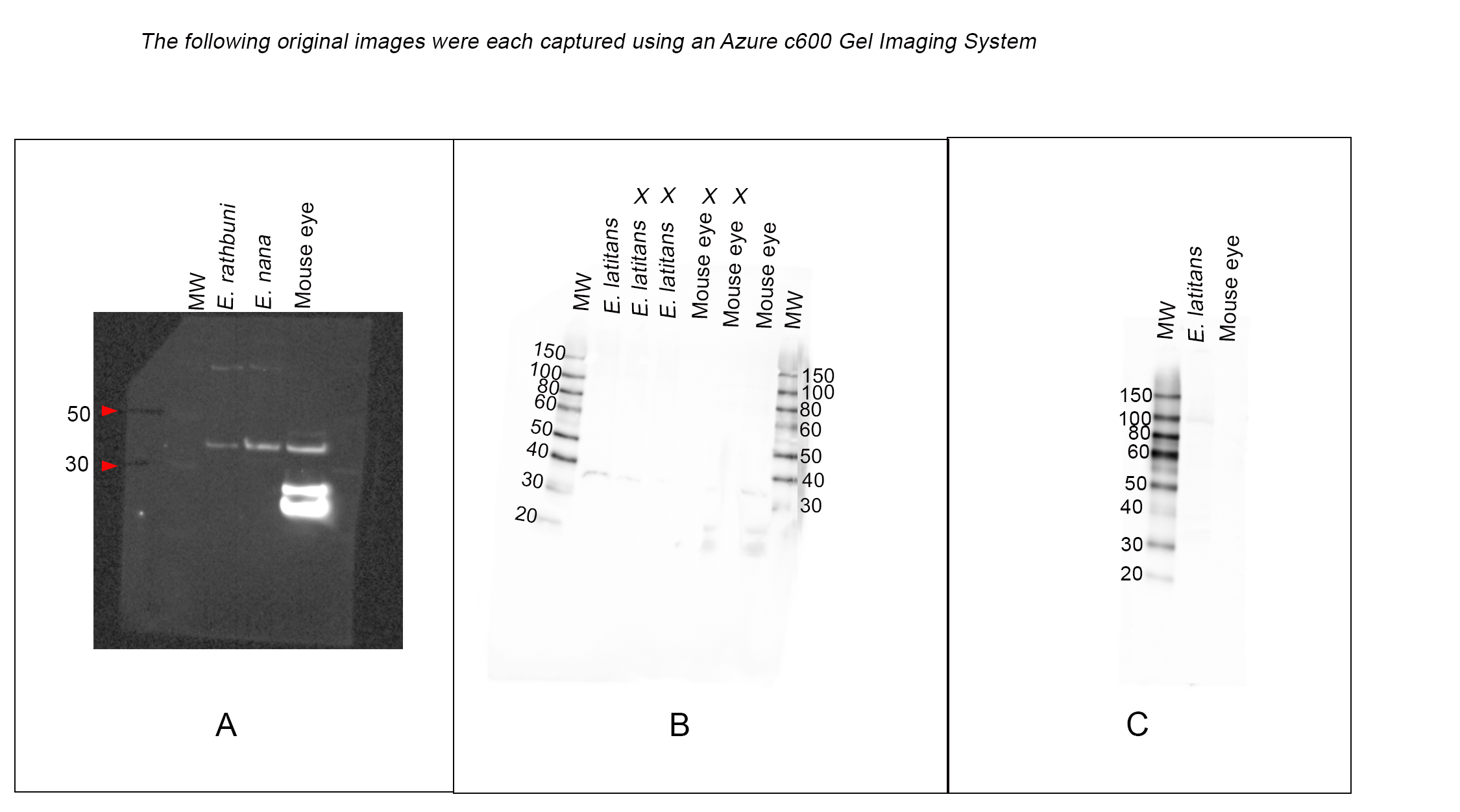

Supplement: S5 Fig — Raw images used to construct S4 Fig. “X” signifies lanes that were cropped in the construction of S4 Fig, in which their former location is indicated by a vertical, dashed line. The image in [A] is inverted in S4A. Western blots were prepared as follows: Tissue lysates were obtained from embryos of E. rathbuni, E. nana, and E. latitans as well as adult mouse eye to serve as a positive control. PagerRuler Plus (Thermo Fisher, cat #26619) [A] and SuperSignal (Thermo Fisher, cat # 84785) molecular weight markers [B,C] were used to estimate the molecular weight of labeled bands. A western analysis using our primary PAX6 antibody suggests that the antibody labels a protein of about 36 kDa in all four taxa. (A) Arrowheads are overlaid on each triangular notch cut into the membrane. E. rathbuni, E. nana, and mouse eye lysates have one labeled band in common at around 36 kDa. A broad, labeled region can be seen in the mouse eye lane between 20 kDa and 27 kDa. (B) At the highest lysate concentrations in a dilution series, E. latitans and mouse eye have one labelled band in common at around 36 kDa. (C) Preadsorbed antibody does not bind to the 36 kDa protein from E. latitans or mouse eye. Labeling of the lower molecular weight bands in mouse eye is also absent following preadsorption of the primary antibody. (TIF) [file pone.0293163.s005.tif]
