# Supplementary material for: PAX6 protein in neuromasts of the lateral line system of salamanders (Eurycea)
Source: PLoS One. 2024 Aug 30;19(8):e0293163. doi: 10.1371/journal.pone.0293163 (PMC11364236; doi:10.1371/journal.pone.0293163)
Supplement: Attachment — Submitted filename: Response to Reviewers.docx [file pone.0293163.s006.docx]

Dear Dr. Klymkowsky:	May 27, 2024

I am grateful for the opportunity to submit the attached the revised manuscript entitled “**PAX6 Protein in Neuromasts of the Lateral Line System of Salamanders (*Eurycea*)**” by Brittany A. Dobbins, Ruben U. Tovar, Braden J. Oddo, Christian G. Teague, Nisa Sindhi, Thomas J. Devitt, David M. Hillis and me (Dana M. García). In this revision, we have sought to address the reviewers’ concerns as I describe point by point below.

*Response to editor’s summary*

Although our research program overall addresses questions relating to evolutionary divergence and the molecular changes that lead to different phenotypic outcomes, the main point of this paper is the appearance of PAX6 in the apical appendages of the hair cells of the mechanosensory neuromasts. Naturally, we are interested in following PAX6 localization during development; this question is addressed in a separate manuscript currently in preparation.

Given the long evolutionary divergence time between amphibians and mammals, we also were concerned about whether the anti-PAX6 antibody raised against mouse PAX6 would recognize salamander PAX6. The salamanders we study are not model organisms; their transcripts and gene sequences are not currently represented in a publicly accessible database. At the time the original manuscript was submitted, we did not have any sequence data for *Pax6* from either the San Marcos salamander (*Eurycea nana*) or the Texas blind salamander (*E. rathbuni*). We now have a partial sequence for *E. nana*; however, we do not yet have probes for *in situ* hybridization. We have, however, been able to do the immunolabeling experiments using antibodies pre-absorbed with the immunogenic sequence published by the manufacturer as suggested. Furthermore, we have run new westerns, also using preadsorbed antibodies. Because embryos for the endangered species originally under investigation were not available, we used a Cascade Caverns salamander (*Eurycea latitans*), a closely related species for these experiments. The new micrographs and blots can be found in the Supporting Information.

*Point by point response to the reviewers’ comments (copied from e-mail with our responses bulleted)*

Reviewer #1: This is a short research note that documents an interesting observation that the Pax6 transcription factor protein appears (at level of immunohistochemistry) to localise to neuromasts in two species of paedomorphic salamanders in the genus Eurycea. The protein is localised to the cytoplasm/cytoskeletal elements of the cells rather than the nucleus, suggesting a role different from gene regulation.
The authors have gone to some trouble to show, through no-primary-antibody controls, western blot and sequence homology comparisons, that the antibody labelling is likely to be specific for Pax6 and not another Pax protein.
I have the following comments. Essentially, while the observation is clearly presented, the data are rather thin and selective:
1) Please provide a more convincing and properly described western blot in FigS1. Needs size markers and a better image. This doesn't look like a western.

- The image of the original western has been reformatted to better represent its appearance. Additional westerns have also been included to address the question of specificity of the antibody by pre-adsorbing the primary antibody prior to its application to control westerns. While the original western used standard protein molecular weight markers (invisible to our imaging system, necessitating notching the membrane to mark the location of the 50 kDa and 30 kDa bands), the new westerns employ molecular weight markers designed for use with chemiluminescence. Both are now presented in S4 Fig.
- A description of the methods by which the westerns were obtained has been added to “Materials and Methods.”

2) Weak cytoplasmic labelling in cells surrounding the neuromasts is observed - please clarify/show whether this persists in skin epidermis away from the neuromasts.

- For the revised manuscript, we re-imaged the larvae using lower laser settings. Our earlier images were prepared using somewhat high (3%) laser power, and we think that may have given the false impression of more widespread labeling than may be biologically relevant. Labeling persists in the apical appendages of hair cells in the new images acquired at 0.67% laser power; however, the weak cytoplasmic labeling in cells surrounding the neuromasts is not detected. A low magnification image (S1 Fig) including a labeled neuromast was obtained using a 20X (0.75 NA) lens and shows more of the surrounding tissue.

3) A much better and more convincing control would be to preabsorb/mix the antibody with the peptide against which it was raised, before addition to tissues. Without this and/or another assay (e.g. in situ or an antibody that recognises a different Pax6 epitope) to show expression in and around neuromasts, there remains some doubt as to whether the staining pattern is real.

- The requested controls have been done, and as mentioned above, extended to western analyses. Preadsorbing the antibody with the peptide published by the manufacturer as the immunogen eliminated labeling in the apical processes and cytoplasm (S3 Fig B).
- In the western analysis, preadsorption eliminated labeling of co-migrating bands at 36 kDa in tissue homogenates obtained from *Eurycea latitans* and mouse eye. It also eliminated lower molecular weight bands observed in the mouse eye homogenate. A faint band migrating at 100 kDa persists in the *E. latitans* homogenate, but because the immunolabeling evident by confocal disappears, we do not think this band accounts for the labeling apparent in the apical appendages.

4) Cytoplasmic localisation of transcription factors proteins is a widespread and well documented phenomenon and cytoplasmic/nuclear shuffling is a mainstream method of activity control (not just for Pax6). This should be discussed.

- We have added to the discussion examples of transcription factors (steroid hormone receptors and heat shock factor 2) translocating between the cytoplasm and nucleus. The example of HSF2 was particular interesting because of its postulated role in forming cytoplasmic bridges between cells of the testis.

5) The authors should possibly also mention Pax6 expression in vertebrate salivary glands, which from figures also appears to be cytoplasmic e.g. <https://karger.com/cto/article/170/2-3/83/90290/Embryonic-Submandibular-Gland-Morphogenesis-Stage>

- We thank the reviewer for directing our attention to this paper. We now mention this paper in our introduction as follows:

In addition to its localization in eyes, including in limbal epithelial niche cells [1], PAX6 expression has been reported in the central nervous system, pancreas, and testes (see [2]), Merkel cells in whisker follicles [3], developing nasal epithelium [4], developing cells of the inner ear [5], and the embryonic submandibular salivary gland [6].

6) Line 28. The limbal epithelial niche is also the eye, not 'in addition' to the eye.

- We restructured the sentence to convey this fact. Please see above.

Reviewer #2: The authors report an observation that Pax6 may be expressed in the cytoplasm of lateral line neuromasts in San Marcos and Texas blind salamander larvae. After reading the manuscript, I am not fully convinced by the specificity of the staining. In addition, the study is very superficial and falls short of providing a relatively detailed description of Pax6 expression in neuromasts.

1. The specificity of Pax6 staining in neuromasts needs to be more rigorously controlled. In addition to omitting the primary antibody, non-immune serum or isotype control should be used in immunofluorescence experiments.

- Please see above response to the editor’s summary and Reviewer 1’s comment 3.

2. In situ hybridization should be performed to confirm Pax6 expression in neuromasts.

- Please see above response to the editor’s summary

3. The authors did not explain in detail the localization of Pax6 in neuromasts. In particular, how it is localized in hair cells versus supporting cells.

- We have reimaged the samples, and now include the following text to explain the localization of the PAX6-labeling:

Immunolabeling with anti-PAX6 antibodies revealed labeling of the neuromasts of the San Marcos salamander and the Texas blind salamander. The labeling was particularly intense in the apical appendages of these cells (Fig. 1). This pattern of labeling was evident in five of the ten neuromasts observed in the Texas blind salamander, and in ten of the eleven neuromasts observed in the San Marcos salamander. At higher laser power (3.03%), fluorescence was also observed in the perinuclear cytoplasm of hair cells; however, at the lower laser power (0.69%) that still revealed apical appendages, labeling of the perinuclear cytoplasm was faint. Three of ten hair cells from the Texas blind salamander showed labeling in the perinuclear cytoplasm; none of the hair cells from the San Marcos salamander showed perinuclear labeling. Labeling within the nucleus of neuromast cells was not observed, nor was labeling in the other cells of the neuromasts or surrounding epithelium (Fig 1 and S1 Fig). PAX6-labeling was also observed in the apical appendages of hair cells in the neuromasts of adult, Texas blind salamander (S2 Fig).

4. The study is very superficial. The authors only examined Pax6 expression in neuromasts at one stage of development. They should perform immunostaining at different stages of neuromast formation.

- As mentioned in the response to the editor’s summary above, we are interested in questions of the evolutionary divergence of developmental processes that lead to different phenotypes; however, in this paper we sought to highlight an interesting aspect of cell biology – the novel localization of PAX6 in association with apical appendages of hair cells in developing as well as adult salamanders– at least in the case of the adult Texas blind salamander. Our hope is that this finding will pique the interest of other scientists interested in cytoskeleton-associated proteins and other questions of cell and neurobiology. We have not yet examined early stage embryos to test whether PAX6 can be found in neuromasts at earlier stages of development. We do not currently have access to earlier stages; however, we think the question of when PAX6 appears in the hair cells is an interesting one, and one which we hope to pursue.

5. Figure 2 is problematic. No San Marcos and Texas blind salamander Pax6 sequences are included in the alignment. It is unclear whether Marcos and Texas blind salamander Pax6 also contain the same immunogenic sequence.

- At the time the manuscript was submitted, no sequence data was available for these species of salamander. During the period of revision, we have gained access to a partial sequence for the San Marcos salamander; however, we still do not have sequence for the Texas blind salamander. The partial sequence inferred from transcriptomic data is now included in the revised Fig 2, and the methodology for acquiring the sequence in included in the Materials and Methods section.

6. There is no protein markers in Figure S2, and size of proteins bands is not clear. In addition, an antibody that can be used for western blot does not necessarily work in immunofluorescence. The authors also do not explain how the labeled protein bands are visualized.

- The western blots are now in a revised S4 Fig, and the methods for obtaining them are now described. In brief, the molecular weight markers in the original western were prestained and visible to the naked eye, but not to the imaging system used to detect chemiluminescence. Therefore, the nitrocellulose membranes were marked by cutting, and in the revised figure, triangles are placed where the cuts were.
- For the preadsorption study also included in the revised figure, molecular weight markers adaptable to that purpose were used and can be seen directly in the figure.

We are grateful for the constructive criticism made by the editor and reviewers, and we hope that you will find the revised manuscript, though still brief, much improved by your input.

As corresponding author, I confirm that the manuscript has been read and approved for submission by all the named authors.

Sincerely,

Dana M. García, Ph. D.

Professor of Biology
