# Supplementary material for: PAX6 protein in neuromasts of the lateral line system of salamanders (Eurycea)
Source: PLoS One. 2024 Aug 30;19(8):e0293163. doi: 10.1371/journal.pone.0293163 (PMC11364236; doi:10.1371/journal.pone.0293163)
Supplement: Attachment — Submitted filename: Response to ReviewersR2.docx [file pone.0293163.s007.docx]

Dear Dr. Klymkowsky:	June 20, 2024

I submit the attached the revised manuscript entitled “**PAX6 Protein in Neuromasts of the Lateral Line System of Salamanders (*Eurycea*)**” by Brittany A. Dobbins, Ruben U. Tovar, Braden J. Oddo, Christian G. Teague, Nisa Sindhi, Thomas J. Devitt, David M. Hillis and me (Dana M. García). We are grateful for the rapid turnaround of the revised manuscript and appreciate the careful review given by you and the reviewers. We seek to address reviewer #2’s concerns below.

*Point by point response to the reviewers’ comments (copied from e-mail with some omissions and with our responses bulleted)*

Reviewer #1: All comments have been addressed.

- Thank you, Dr. Collinson, for your previous comments. We are pleased that you found our responses satisfactory.

Is the manuscript presented in an intelligible fashion and written in standard English?

PLOS ONE does not copyedit accepted manuscripts, so the language in submitted articles must be clear, correct, and unambiguous. Any typographical or grammatical errors should be corrected at revision, so please note any specific errors here.

Reviewer #1: Yes

Reviewer #2: No

- We have carefully read the manuscript, searching for misspellings and grammatical errors, taking advantage of the tools available through Microsoft Word. We identified an error in which we had twice cited a reference in a single sentence (line 247), and we corrected that error. We also changed the wording on the description of hair cells (lines 314-316) to better describe apical appendages in the context of multiple cells. Without further guidance, we are unsure how to address this comment.

Reviewer #2: The manuscript presents only a few images showing possible cytoplasmic localization Pax6 in neuromast hair cells of salamander larvae at one single stage. I am not sure this is a sufficiently novel and complete study to warrant publication. It is necessary to more comprehensively address the appearance of Pax6 in the apical appendages of hair cells. If no embryos could be obtained from these species for a comprehensive analysis of Pax6 localization in hair cells, the authors may use other amphibians or even fish to perform the study. This will help understand whether the cytoplasmic localization of Pax6 in hair cells is conserved.

- We agree with the reviewer that the manuscript is not extensive; however, PLOS ONE guidelines state that the journal accepts “all rigorous research.” While the web site does not define rigor, a common understanding involves the application of appropriate research methodology and controls to meet stated objectives of the research. Thanks in part to the additional controls proposed by the reviewers, we believe have met the standard for rigor and that we are conservative in our interpretation of the results and conclusions that we draw, specifically that PAX6 localizes to the apical appendages of hair cells of neuromasts, a novel finding that has not been previously reported. Additionally, our findings in Texas blind salamander (*Eurycea rathbuni*) include an adult specimen (S2 Fig). Furthermore, although our study system is limited to salamanders of the genus *Eurycea*, we do show this localization in two different species representing very different ecotypes, indicating some level of conservation during development. Extending the observation to other taxa may or may not reveal conservation; however, the point of this study is simply to report the novel observation of PAX6 localization in the apical appendages of hair cells.
- We also agree that it is necessary to do more to comprehensively address the appearance of PAX6 in hair cells, and we are hopeful that by publishing this paper, we will open the door to other investigators, each with a unique perspective on the implications of this finding and the appropriate follow-up experiments. For our part, we are hoping to pursue the question of whether PAX6 is associated with the cytoskeleton in this system. Our laboratory has no full-time researchers, so we anticipate the study will take at least two years to complete.

Additionally, we have carefully checked and cross-checked all references, and we used “Retraction Watch” to see whether any had been retracted. We found no evidence that any had.

We are grateful for the constructive criticism and the effort expended to review our manuscript. We hope this rebuttal satisfies the concerns raised by the reviewer.

As corresponding author, I confirm that the manuscript has been read and approved for submission by all the named authors.

Sincerely,

Dana M. García, Ph. D.

Professor of Biology
