# Supplementary material for: PAX6 protein in neuromasts of the lateral line system of salamanders (Eurycea)
Source: PLoS One. 2024 Aug 30;19(8):e0293163. doi: 10.1371/journal.pone.0293163 (PMC11364236; doi:10.1371/journal.pone.0293163)
Supplement: Attachment — Submitted filename: Response to ReviewersR3.docx [file pone.0293163.s008.docx]

Dear Dr. Abo-Al-Ela:	August 13, 2024

I submit the attached the revised manuscript entitled “**PAX6 Protein in Neuromasts of the Lateral Line System of Salamanders (*Eurycea*)**” by Brittany A. Dobbins, Ruben U. Tovar, Braden J. Oddo, Christian G. Teague, Nisa Sindhi, Thomas J. Devitt, David M. Hillis and me (Dana M. García). We are grateful for the rapid turnaround of the revised manuscript and appreciate the careful review of the second revision given by you and taking into account the reviewers’ previous comments.

*Point by point response to the academic editor’s and reviewer’s comments (copied from e-mail with some omissions and with our responses bulleted)*

Academic Editor: Due to several limitations – such as the unavailability of genome sequences, the difficulty in obtaining embryos from the endangered Eurycea, and the inability of the authors to perform additional confirmatory methods – it is crucial to clearly outline these limitations in the manuscript. This information should be included in relevant sections, including the abstract. I strongly recommend using probabilistic language where appropriate when reporting results throughout the manuscript and suggesting further investigatory studies to confirm or refute these findings.

- The authors acknowledge the limitations of the study and have attempted to address the editor’s comments and previous reviewers’ comments as detailed below in the order items appear in the manuscript (Line numbers are based on the unmarked manuscript):
  - To speak probabilistically, in line 18 (Abstract): “…PAX6 … appears to be present in the mechanosensory neuromasts…” and in line 22 (Abstract): “anti-PAX6 antibody labeled structures that were extranuclear….”
  - To further communicate the difficulty of obtaining samples, in line 19-22 (Abstract): “…Using immunohistochemistry and confocal microscopy to examine a limited number of larvae of two species, listed by the United States of America’s federal government as threatened (*E. nana*) or endangered (*E. rathbuni*)….”
  - To communicate the unavailability of genomic or other sequence data, in line 181-182 (Methods): “Sequence data are not publicly available for salamanders in the genus *Eurycea*.”
    - We note that in R2, we were able to add a partial sequence of *Pax6* obtained from *Eurycea nana* and that result is shown in Figure 2.
  - To communicate the prevalence and extent of labeling and better flesh out the certitude of our observation (probabilistic language), we reevaluated our images, counted all neuromasts observed and described the labeling in lines 193-201 (Results).
    - We added to this section information about the number of neuromasts in which labeling was observed in the apical appendages as well as the number of individuals from each of the two species. In short, we did not observe labeling in every neuromast; however, we did observe labeling of the apical appendages in every individual salamander examined.
    - For R2, we had added in preadsorption experiments for both immunohistochemical and western analyses. When antibodies were preadsorbed in both cases, labeling was reduced below detection, corroborating our inference that the labeling we report reveals the location of PAX6.
  - To further emphasize the limitations of the study, we conclude the Discussion with the paragraph that follows (lines 381 – 390):

A challenge of extending the work we present here is the availability of specimens of these understudied, threatened and endangered species for which publicly available genomic data is not available. Work is underway to generate transcriptomic data, and preliminary results for the sequence of *Pax6* from the transcriptome of *Eurycea* *nana* are included herein. We expect to further investigate *Pax6* expression in skin, and more precisely in neuromasts in future studies, contingent on the availability of salamander larvae. The immunohistochemical results we report here are nevertheless tantalizing, and though the number of individuals probed with anti-PAX6 antibodies is small, it is worth noting that labeling of the apical appendages of hair cells in the neuromasts was observed in all of them. That it was not observed in all neuromasts could be attributed to a failure to capture the apical appendages in some tissue sections.

Reviewer #1: All comments have been addressed.

- Thank you, Dr. Collinson, for your previous comments. We are pleased that you found our responses satisfactory.

Additional changes have been made in hopes of improving the readability of the manuscript. These are not detailed here, but our evident in the version with “Track Changes.”

We have carefully checked and cross-checked all references, and we used “Retraction Watch” to see whether any had been retracted. We found no evidence that any had.

We are grateful for the constructive criticism and the effort expended to review our manuscript. We hope this rebuttal satisfies the concerns raised by the academic editor.

Sincerely,

Dana M. García, Ph. D.

Professor of Biology
